# Surgical

**Published:** 1856-12

**Authors:** 


### SURGICAL.

The following dialogue took place recently in a consultation between Surgeon A. Trobridge, aged seventy-four, and his patient, aged seventy.

The patient had been suffering for several years with necrosis, or diseased bone of his leg; finally death of the whole bone took place, and gangrene of all the soft parts to the knee. The attending physician apprized him of his perilous condition, and he sent for the Surgeon for advice, and the following conversation ensued:

Surgeon—You are a very aged man?

Patient—Yes sir, I am so.

S.—Was your health good in your younger days?

P.—I was very healthy until this leg became diseased, thirteen years since.

S.—What was the matter with your leg at first?

P.—The doctor said it was a disease of the bone.

S.—It was undoubtedly, and has brought you near to death.

P.—Yes sir, and I have sent for you to advise me what to do.

S.—Do you wish to live one or two years longer?

P.—I do. I have a small place, and two daughters; one keeps school and the other keeps my house and cares for me. We are poor, but live comfortably by our industry and economy. I should like to live a little longer on their account.

S.—Your only chance is the immediate amputation of your limb.

P.—I am sensible of that, and will submit to the operation. Will you use chloroform?

S.—I use chloroform in many cases, but have established as a principle not to use it on an old person or a very young one. I ask of you only two minutes' suffering for the operation.

P.—O, I can suffer any pain two minutes.

S.—We are then agreed without further loss of time. The operation was made within that time, and the patient recovered, and still lives with his daughters.

Daughters—What is your bill, doctor?

S.—Keep enough to take good care of your father, make yourselves comfortable, and pay me what you can.

ELLISBURGH.

Here was decision and benevolence on one part, and a fortitude, confidence and submission on the other, very important on such occasions.—*American Medical Gazette.*

